# Lycopene Aggravates Acute Gastric Injury Induced by Ethanol

**DOI:** 10.3389/fnut.2021.697879

**Published:** 2021-08-17

**Authors:** Xin Chen, Yuechao Zhao, Keying Liu, Zexu Li, Xingru Tan, Yulong Wang, Na Gao, Chenming Liu, Xiaoqi Fang, Yanlong Wang

**Affiliations:** ^1^Collaborative Innovation Center for Birth Defect Research and Transformation of Shandong Province, Jining Medical University, Jining, China; ^2^College of Teacher Education, Qilu Normal University, Jinan, China; ^3^Amicogen (China) Biopharm Company, Jining, China; ^4^School of Public Health, Shandong First Medical University & Shandong Academy of Medical Sciences, Taian, China

**Keywords:** lycopene, ethanol, acute gastric injury, antioxidant, liver

## Abstract

Lycopene is an important natural red pigment with strong singlet oxygen and peroxide free radical quenching ability. Ethanol directly destroys the epithelial cells of gastric mucosa, causing oxidative damage and inflammation. To evaluate the effect of lycopene on the ethanol induced gastric injury, 112 adult male Kunming mice were randomly divided into normal control, lycopene control, gastric injury control, omeprazole (20 mg/kg) positive control, and lycopene experimental groups (at doses of 10, 50, 100, and 150 mg/kg body weight) in this study. The general and pathological evaluation, gastric secretion, as well as the levels of antioxidant and inflammatory factors were detected. In lycopene experimental groups, the amount of gastric juice were lower than that in the gastric injury control group; the levels of T-SOD, and the levels of MDA and inflammatory factors (MMP-9 and MCP-1) decreased. However, general and pathological evaluation of gastric tissues revealed that lycopene (especially at high doses) could aggravate acute gastric mucosal injury induced by ethanol. Therefore, lycopene (especially at high doses) aggravates acute gastric mucosal injury caused by ethanol, but this was not due to oxidative stress or inflammatory factors. In lycopene control group, the levels of MTL, T-SOD, and NO increased, but the levels of ALT and AST decreased, indicating that lycopene has a protective effect on the stomach and liver when ethanol wasn't taken. It reminds us that, when alcohol is consumed in large quantities, consumption of lycopene products should be carefully considered.

## Introduction

Acute gastric injury caused by ethanol is a common clinical disease, especially in males. The integrity of the stomach is mostly maintained by pre-epithelial factors (mucus, bicarbonate, and phospholipids), epithelial factors (prostaglandins, heat shock proteins, trefoil factor family peptides, and enterotoxins), and post-epithelial factors (nitric oxide (NO) and prostacyclin) (1). These defense mechanisms can protect the gastric mucosa from local damage and maintain its structural and functional integrity (2). Ethanol directly destroys the integrity of the gastric mucosal barrier, increases the permeability of the mucosa, and damages gastric acid cells, causing gastric mucosal damage and bleeding (3, 4). When the gastric mucosa is damaged, pro-inflammatory cytokines become unbalanced, and neutrophils deform and migrate to the damaged site, increasing the concentration of reactive oxygen species (ROS) and other inflammatory mediators, leading to oxidative damage (5, 6). Therefore, oxidative stress plays an important role in alcohol-induced gastric mucosal damage. There is a variety of endogenous antioxidant enzymes in gastric cells, such as superoxide dismutase (SOD) and catalase (CAT), which can maintain gastric homeostasis by scavenging ROS. SOD catalyzes the disproportionation of O^2−^ into H_2_O_2_ Furthermore, CAT accelerates the decomposition of H_2_O_2_ into water and oxygen (7). Therefore, endogenous antioxidant enzymes play an important role in protecting the integrity of the gastric mucosa.

Lycopene is one kind of important pigment carotenoids. It is a fat-soluble linear and highly unsaturated polyolefin compound composed of 11 hydrocarbons-conjugated double bonds and 2 hydrocarbon/non-conjugated double bonds, it has anti-cancer, anti-obesity, and improving immunity functions and it protects the cardiovascular system (8–11). The ability of lycopene to quench singlet oxygen is more than twice that of β-carotene and 100 times that of vitamin E (12). Boyacioglu et al. found that lycopene had a protective effect against indomethacin-induced gastric ulcer and oxidative stress in rats (13). Lycopene has the strongest protective effect at an additive dose of 100 mg/kg. Jang et al. found that lycopene inhibited *Helicobacter pylori*-induced human gastric adenocarcinoma cell line AGS cells increases in ROS, apoptosis, cell cycle distribution changes, double-strand DNA breaks, ataxia-telangiectasia-mutated (ATM), and ATM-Rad3-related DNA damage response (14). Therefore, it is beneficial for the treatment of gastric diseases related to DNA oxidative damage caused by *Helicobacter pylori*.

The effect of lycopene on ethanol-induced acute gastric mucosal damage has not been well studied. This study analyzed the effect of lycopene on the acute gastric injury induced by ethanol in mice through visual observation, pathological analysis, and biochemical index detection, in order to provide a reference for the use of lycopene in clinical and daily drinking.

## Materials and Methods

### Reagents

Lycopene, Omeprazole (OME), 1×PBS (phosphate buffered saline) were purchased from Beijing solarbio science and technology Co., Ltd. (Beijing, China). Reagents such as absolute ethanol, phenolphthalein, NaOH, NaCl and corn oil were obtained from Sinopharm Chemical Reagent Co., Ltd. (Shanghai, China). These chemical reagents were of analytical grade. CAT detection kit (Cat. No. A007-2-1), NO detection kit (Cat. No. A013-2-1), malondialdehyde (MDA) detection kit (Cat. No. A003-1-2) and serum total superoxide dismutase (T-SOD) detection kit (Cat. No. A001-1-2) were purchased from Nanjing Jiancheng Bioengineering Institute (Nanjing, China) (15), and the Enhanced BAC protein assay kit (Cat. No. P0010S) was purchased from Beyotime Biotechnology (Shanghai, China) (16). Enzyme-linked immunosorbent assay (ELISA) kits, including mouse Alanine aminotransferase (ALT) ELISA kit (Cat. No. ml063179-2), aspartate aminotransferase (AST) ELISA kit (Cat. No. ml058659-2), Matrix metalloproteinase-9 (MMP-9) ELISA kit (Cat. No. ml037717-2), monocyte chemotactic factor-1 (MCP-1) ELISA kit (Cat. No. ml037840-2), and motilin (MTL) ELISA kit (Cat. No. ml201829-2) were purchased from Shanghai enzyme-linked biotechnology Co., Ltd. (Mlbio, Shanghai, China) (17).

### Animals

A total of 112 adult specific pathogen-free male Kunming (KM) mice (6 weeks-old and weighing 27–32 g) were obtained from Jinan Pengyue laboratory animal Brewing Co., Ltd. (Jinan, China). These mice were suspended in a cage with wire mesh bottom at 20–25°C to prevent fecal eating, and allowed free access to tap water and standard laboratory chow diet in an animal room with 12 h/12 h light/dark illumination cycle.

### Experimental Protocol and Establishment of an Ethanol-Induced Gastric Lesions Model

After a 1-week adaptation period to the environment, eight groups of mice were assigned (*n* = 14 for each group). These groups were arranged as follows: normal control group (NC), lycopene control group (LYC), gastric injury control group (IC), omeprazole control group (OMEC), and four lycopene experimental groups. The lycopene experimental groups include ultralow-dose group (LYC-UL), low-dose group (LYC-L), medium-dose group (LYC-M), and high-dose group (LYC-H). All mice were raised under standard control conditions, fasted for 24 hours before ethanol gavage, and had free access to tap water. In addition, all drugs were administered once daily for 4 days and which were given by gastric gavage. Lycopene was suspended in corn oil and administered at four dose (10, 50, 100, and 150 mg/kg body weight) (13). Omeprazole (20 mg/kg body weight) was given only to the OMEC group. On the last day of treatment, 6.67 ml/kg body weight of absolute ethanol (18) was given to each mouse in IC, OMEC, LYC-UL, LYC-L, LYC-M, and LYC-H groups after 1 hour of intragastric administration. The NC and LYC groups were received double-distilled water in specified volume.

### Sample Collection

One hour after administration of absolute ethanol/double-distilled water, the retro-orbital blood was collected. Then, all mice were sacrificed. And their stomachs and livers were immediately removed. The stomachs were opened along the greater curvature and the contents were collected. After cleaned with normal saline, the injury occurrence in gastric mucosal layer was carefully observed. Each gastric tissue sample was cut into two parts; one part was embedded in OCT compound (Tissue-Tek) for frozen section and immunohistochemistry, another part was frozen in liquid nitrogen. Serum, stomach, and liver of these mice were stored at −80°C for biochemical analysis.

### Determination of Total Acidity of Gastric Juice

The contents of stomach were collected from stomach. Then, these contents were placed for centrifugation at 3,000 rpm for 10 min at 4°C. The total volume of gastric juice and the total acidity of gastric juice were detected (19). The total acidity of gastric juice was evaluated by titration using a 10 mmol NaOH solution and phenolphthalein as indicator.

### Gastroprotective Assessments

The removed gastric tissues were immediately rinsed with 4°C normal saline to wash away the blood. Then, the gastric mucosal lesions were measured using a visual inspection. Image J image processing software (NIH, USA) was used to select and measure the total area of gastric mucosa as well as gastric injury area. The gastric injury was manifested as an elongated band of hemorrhagic lesions. Then, the gastric injury index (18) and gastric inhibition rate (19) were calculated. Gastric injury index = gastric injury area of each mouse/total gastric mucosal area of each mouse × 100. Gastric injury inhibition rate (%) = (injury index of IC group – injury index of treatment groups)/injury index of the IC group.

### Determination of NO, MDA, and T-SOD Levels in Serum

Retro-orbital blood was collected and then centrifuged at 3,000 rpm for 10 min to acquire the serum. The levels of NO were detected using a NO detection kit. 10 ul serum was taken and added to reagent I as well as reagent II. The mixture was stirred for 15 min, and centrifuged at 4°C and 4,500 rpm for 10 min to acquire the supernatant. The supernatant, blank (equal amount of double-distilled water) and standard (20 umol/L sodium nitrite), were taken and mixed with color developer. Then, standing by 15 min and the absorbance value at 550 nm was detected by microplate reader. The levels of MDA were detected using a MDA detection kit and the absorbance value at 532 nm was detected. The contents of MDA were calculated based on the standard tetraethoxypropane. Superoxide dismutase (SOD) has been shown to play an important role in the oxidative and antioxidant balance of the organism. Therefore, the levels of T-SOD were detected using a T-SOD detection kit (hydroxylamine method) by the absorbance value at 550 nm.

### Determination of MCP-1, MMP-9, and Motilin Levels in Gastric Tissues

Gastric injured tissues were immersed in ice-cold phosphate buffered saline (containing protease inhibitors) for homogenization and further crushed by using a Scientz-IID sonifier (Ningbo Scientz Biotechnology Co., China). Then, these tissue mixtures were centrifuged at 4°C and 4,500 rpm for 10 min, and the supernatant was collected as crude enzyme of gastric tissues. Three kinds of ELISA kits, including MCP-1, MMP-9, and Motilin were equilibrated at room-temperature (about 25°C) for 30 min, and 50 μl of crude enzyme or standards were added to the appropriate microtiter plate wells. Then, 100 μl of the horseradish peroxidase conjugated anti-MCP-1, anti-MMP-9, and anti-motilin solutions were added to each microplate well and incubated for an hour at 37°C. After washing five times with the assay buffer, 50 μl chromogenic agents A and B were added to each microplate well, shaken gently to mix, and kept in a dark place 15 min at 37°C. Then, the reaction was terminated by adding 50 μl of the stop solution, and the absorbance value at 450 nm wavelength was detected within 15 min. The standard curve was plotted according to the standards and the levels of MCP-1, MMP-9, and motilin were calculated separately.

### Determination of CAT Activity in Liver Tissues

Liver tissues were immersed in ice-cold phosphate buffered saline (containing protease inhibitors) for homogenization and further crushed. Then, these mixtures were centrifuged at 4°C, 4,500 rpm for 10 min, and the supernatants were collected as crude enzyme solution of liver tissues (20). The total protein concentrations of the crude enzyme solutions were detected using a BCA total protein concentration detection kit. Then, the CAT activities of liver tissues were detected using a CAT detection kit (ultraviolet method). The substrates solutions with absorbances between 0.5 and 0.55 were prepared. Then, 5 ul of the diluted crude enzymes were added to the cuvette, and 750 ul of the substrate solutions were speedily flushed into colorimetric ware to measure the absorbance value at 240 nm wavelength before and after 1 min. The CAT activities were calculated based on the total protein concentrations of the sample.

### Determination of ALT and AST Levels in Liver Tissues

And from the liver tissues were measured with ELISA kits. Two kinds of ELISA kits (ALT and AST) were equilibrated at room-temperature for 30 min, and 50 μl of crude enzyme or standards were added to the appropriate microtiter plate wells. Then, 100 μl of the horseradish peroxidase conjugated anti-ALT and anti-AST solutions were added to each microplate well and incubated for an hour. After washing, chromogenic reaction, and termination reaction, the absorbance value at 450 nm wavelength was detected. The standard curve was plotted according to the standards and the levels of ALT and AST were calculated separately. All procedures were performed according to the manual recommended by the manufacturer.

### Histopathological Evaluation

Gastric tissue specimens from the mice which were sectioned at a thickness about 3–4 μm and then stained with hematoxylin and eosin. These samples were examined pathologically in upright fluorescence microscope (ni-u; Nikon Co., Tokyo, Japan). According to the method of Amirshahrokhi (21), we assessed each tissue section for gastric mucosal epithelial injury score: epithelial cell loss (score: 0–3), hemorrhagic damage (score: 0–4), Gastric mucosal epithelial cell loss (score: 0–3), epithelial mucosal edema (score: 0–4), and the presence of inflammatory cells (score: 0–4), yielding a maximum total score of 14. Subsequently, these pathological frozen sections were assessed by two experienced histopathologists who were blinded to the treatment.

### Statistic Analysis

All data were expressed as mean ± standard deviation (SD). Statistical analysis was performed using SPSS 26.0 statistical software (SPSS Inc., Chicago, IL, USA). The statistical significance of any difference in each parameter among the groups was evaluated by one-way analysis of variance (ANOVA) (S-N-K method). *P* < 0.05 was considered statistically significant.

## Results

### The Effect of Lycopene on Ethanol-Induced Gastric Injury

#### General Evaluation of Gastric Tissues

**Figure 1:**
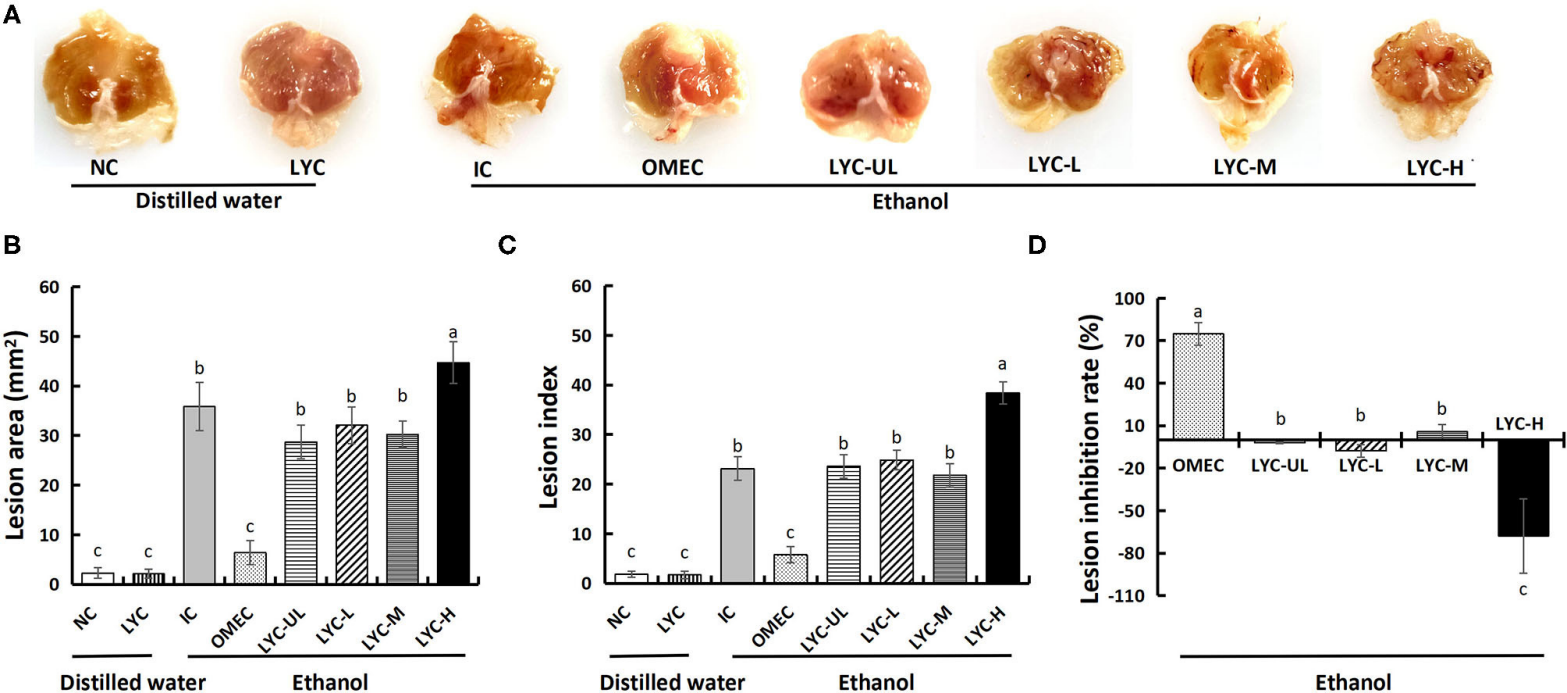
Effect of lycopene on the ethanol-induced gastric lesions in mice. **(A)** Macroscopic appearance of gastric mucosa. NC, normal control; LYC, lycopene control group, 100 mg/kg bw of lycopene; IC, injury control group; OMEC, omeprazole control group, 20 mg/kg bw of omeprazole; LYC-UL, ultralow-dose group, 10 mg/kg bw of lycopene; LYC-L, lycopene low-dose group, 50 mg/kg bw of lycopene; LYC-M, lycopene medium-dose group, 100 mg/kg bw of lycopene; LYC-H, lycopene high dose group, 150 mg/kg bw of lycopene. NC and LYC groups were perfused with distilled water; IC, OMEC, LYC-UL, LYC-L, LYC-M, and LYC-H groups were perfused with 6.67 ml/kg absolute ethanol. **(B)** Gross lesion area. **(C)** Lesion index. **(D)** Lesion inhibition rate. Different letters (a–c) indicate significant differences (*P* < 0.05) between different groups.

Oxidative stress is the main manifestation of absolute ethanol-induced gastric mucosal injury in mice, and lycopene has a protective effect on oxidative stress. In this study, mice were administered absolute ethanol gavage and treated with different doses of lycopene. Through general observation of the gastric mucosa, we found that there were no lesions in the NC and LYC groups, but lesions in IC, OMEC, and lycopene experimental groups (Figure 1A). Among them, the IC group and the LYC-H group had the most severe bands, with extensive bleeding and collapse. Moreover, the gastric mucosal injury area (Figure 1B, Supplementary Figure 1) and injury index (Figure 1C) of the LYC-H group were increased by 25% and 66%, respectively (*P* < 0.05), compared with the IC group. The injury inhibition rate (Figure 1D) of the OMEC group was approximately 75%, while the inhibition rates of the LYC-UL, LYC-L, and LYC-H groups were −2, −8, and −68%, respectively. These results suggest that lycopene aggravates gastric mucosal injury caused by absolute ethanol, especially at high doses.

#### Pathological Evaluation of Gastric Tissues

**Figure 2:**
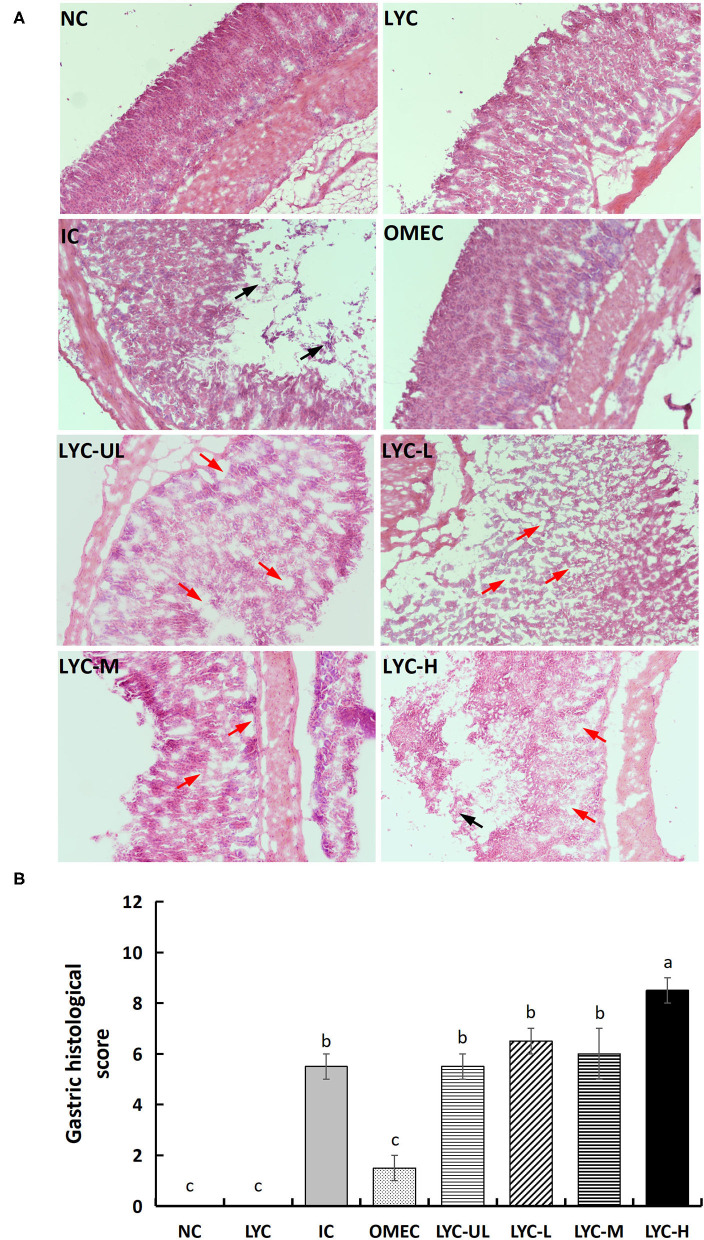
Histological morphology **(A)** and total pathological scores **(B)** of gastric mucosa in mice (magnification 100×). NC, normal control; LYC, lycopene control group; IC, injury control group; OMEC, omeprazole control group; LYC-UL, ultralow-dose group; LYC-L, lycopene low-dose group; LYC-M, lycopene medium-dose group; LYC-H, lycopene high dose group. NC and LYC groups were perfused with distilled water; IC, OMEC, LYC-UL, LYC-L, LYC-M, and LYC-H groups were perfused with 6.67 ml/kg absolute ethanol. Different letters (a–c) indicate significant differences (P < 0.05) between different groups.

The results of pathological analysis of gastric tissue slices are shown in Figure 2A. No histological changes were observed in the gastric specimens of the NC and LYC groups. After treatment with absolute ethanol, the IC and lycopene experimental groups (especially the LYC-H group) showed local necrosis and shedding of gastric mucosal epithelial cells, in which the gastric pit structure also disappeared (black arrow). The lycopene experimental groups (LYC-UL, LYC-L, LYC-M, LYC-H) showed mild oedema in the gastric mucosal layer or enlarged gaps between some of the fundus glands (red arrows). The animal histopathological score results also showed that the absolute ethanol group and the lycopene experimental group had higher scores than others, which meant that the degree of gastric injury was higher than others (Figure 2B). The pathology score of the LYC-H group was about 1.55-fold that of IC group, 1.42-fold that of LYC-M, 1.31-fold that of LYC-L, and 1.55-fold that of LYC-UL. Thus, absolute ethanol can cause severe gastric mucosal damage. At lycopene experimental groups and especially at 150 mg/kg lycopene, the pathological changes in mouse gastric tissue integrity of the mucosal epithelium, and destruction of the glandular structure caused by absolute ethanol are more obvious.

**Figure 3:**
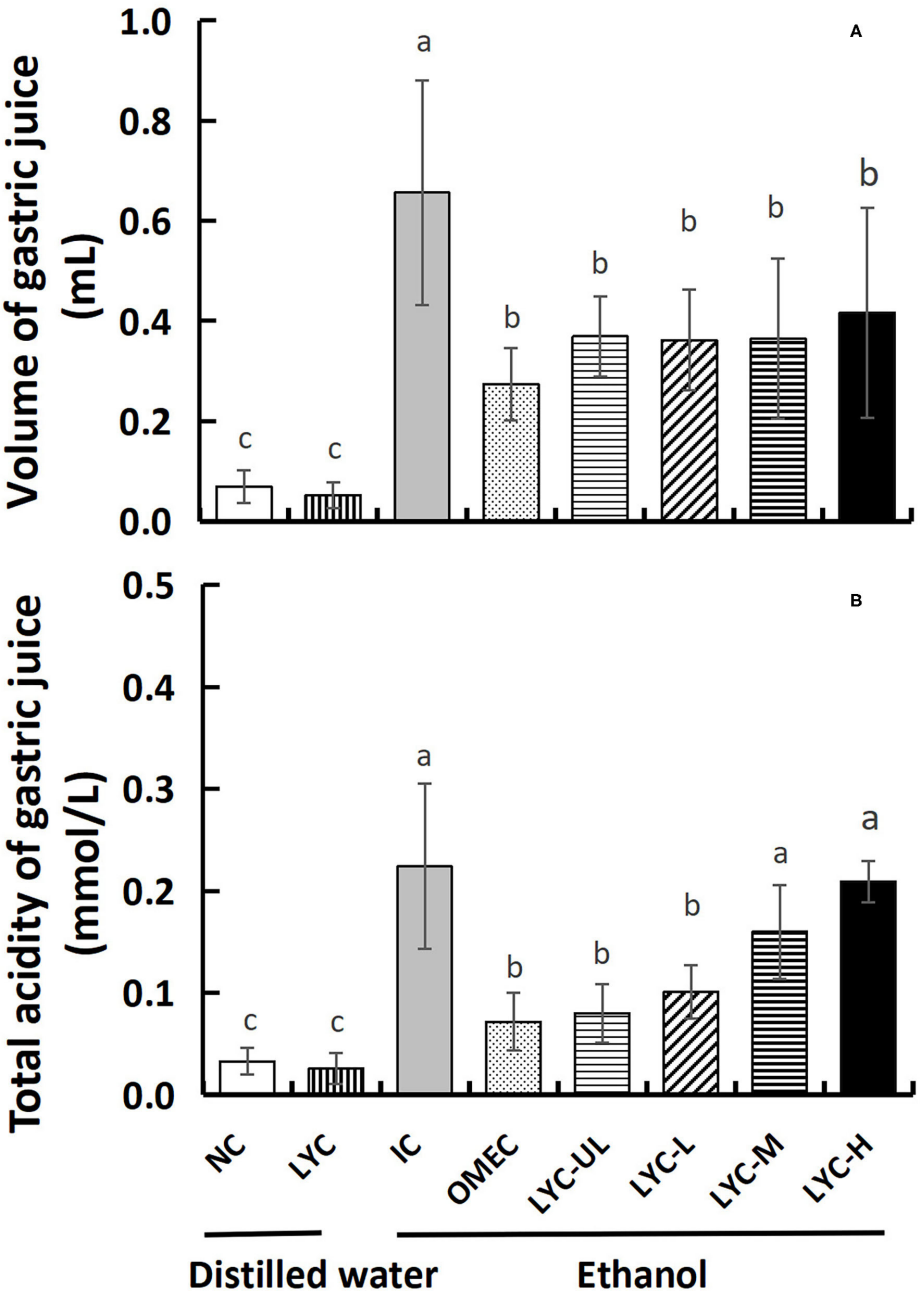
Volume and total acidity of gastric juice. **(A)** Volume of gastric juice. **(B)** Total acidity of gastric juice. NC, normal control; LYC, lycopene control group; IC, injury control group; OMEC, omeprazole control group; LYC-UL, ultralow-dose group; LYC-L, lycopene low-dose group; LYC-M, lycopene medium-dose group; LYC-H, lycopene high dose group. NC and LYC groups were perfused with distilled water; IC, OMEC, LYC-UL, LYC-L, LYC-M, and LYC-H groups were perfused with 6.67 ml/kg absolute ethanol. Different letters (a–c) indicate significant differences (*P* < 0.05) between different groups.

The increase in gastric juice volume and total acidity has been shown to aggravate acute gastric ulcers in mice (22); therefore, we measured the gastric juice volume and total acidity. As shown in Figure 3, the gastric juice volume and total acidity of the NC and LYC groups were lower than those of the other groups, and there was no significant difference between these two groups. After gastric gavage with absolute ethanol, the gastric juice volume and total acidity of the mice in the IC group increased significantly. After gastric gavage with omeprazole and different doses of lycopene (10, 50, 100, and 150 mg/kg), the amount of gastric juice secretion in mice was significantly lower than that in IC group, which decreased by 58, 44, 45, 45, and 37%, respectively, compared with the IC group (Figure 3A). At 100 and 150 mg/kg lycopene, the total acidity was not significantly different from that of the IC group, but it was significantly higher than that in the OMEC group (Figure 3B). Compared with the IC group, the total acidity level of OMEC, LYC-UL, and LYC-L groups were significantly decreased by 68%, 64%, and 55%, respectively (*P* < 0.05). Therefore, low-dose lycopene reduced the total acidity of gastric juice; however, its effect was less than that of omeprazole. Although high-dose lycopene could reduce the volume of gastric juice, it did not reduce the total acidity of gastric juice.

### The Effect of Lycopene on the Levels of MDA, NO, and T-SOD in Serum

**Figure 4:**
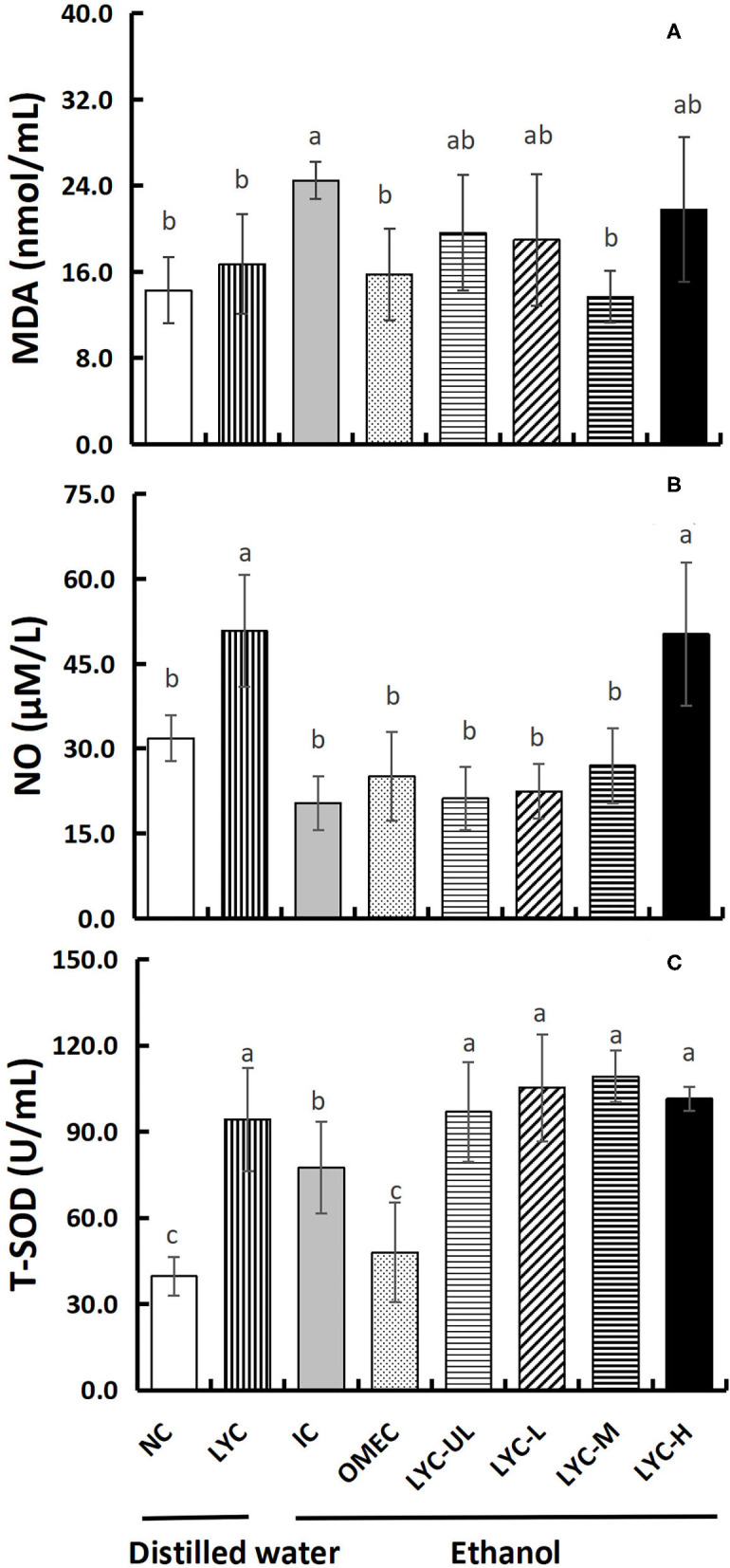
Effect of lycopene on antioxidant factors of serum. NC, normal control group; LYC, lycopene control group; IC, injury control group; OMEC, omeprazole control group; LYC-UL, ultralow-dose group; LYC-L, lycopene low-dose group; LYC-M, lycopene medium-dose group; LYC-H, lycopene high dose group. **(A)** Malonaldehyde (MDA). **(B)** Nitric oxide (NO). **(C)** Total superoxide disumutase (T-SOD). Values are means standard deviation of 14 mice and triplicate measurements. Different letters indicate significant differences (*P* < 0.05) between different groups.

MDA, NO, and SOD are often used to evaluate the antioxidant levels in gastric injury models. Therefore, we tested these three factors in the serum (Figure 4). After absolute ethanol gavage, MDA levels in the IC group were significantly higher than those in the NC and lycopene experimental groups (Figure 4A). The levels of MDA in the OMEC, LYC-UL, LYC-L, LYC-M, and LYC-H groups were 0.64-, 0.80-, 0.77-, 0.56-, and 0.89-fold of those in the IC group, respectively. And there was no significant difference among NC, LYC, OMEC, and LYC-M groups. By detecting the level of NO in the serum, we found that without ethanol treatment, the level of NO in the LYC group was 1.60-fold of those in the NC group (Figure 4B). After ethanol treatment, high dose of lycopene significantly increased the level of NO. In addition, the level of T-SOD in the serum of mice from the lycopene treatment groups were significantly increased, regardless of whether absolute ethanol was added (Figure 4C). Without ethanol treatment, the level of T-SOD in the LYC group was significantly higher than that in the NC group, which was approximately 2.37-fold that of the NC group. After ethanol treatment, the levels of T-SOD in the LYC-UL, LYC-L, LYC-M, and LYC-H groups were 1.25-, 1.36-, 1.41-, and 1.31-fold that of the IC group, respectively. Therefore, regardless of whether treated with ethanol, after supplementation with lycopene, the NO and T-SOD levels in the serum of mice increased significantly. However, the MDA level in lycopene experimental groups were significantly lower than that in the IC group after ethanol treatment.

### The Effect of Lycopene on the Levels of MTL, MCP-1, and MMP-9 in Gastric Tissues

**Figure 5:**
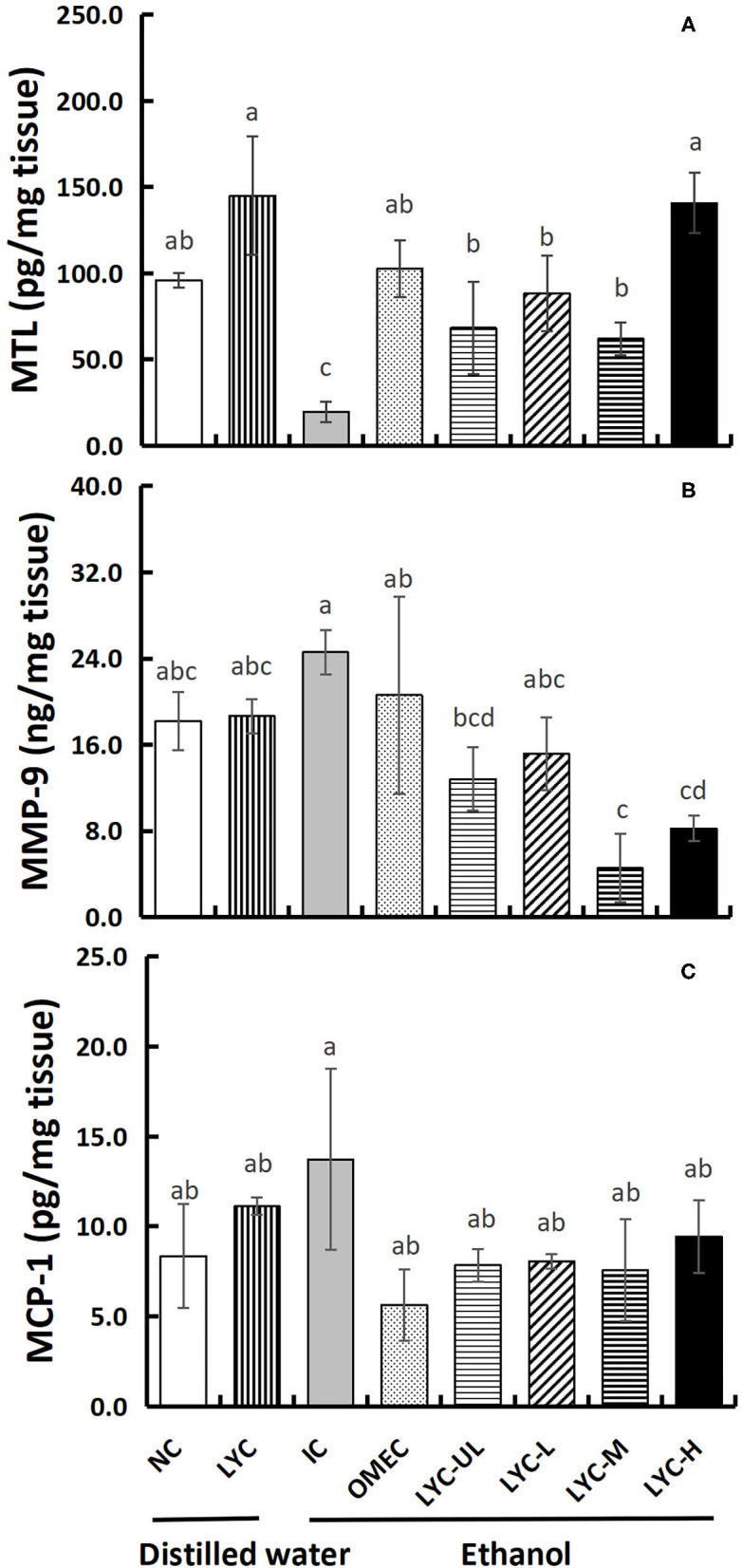
Effect of lycopene on MTL, MMP-9, and MCP-1 levels of gastric tissues. NC, normal control group; LYC, lycopene control group; IC, injury control group; OMEC, omeprazole control group; LYC-UL, ultralow-dose group; LYC-L, lycopene low-dose group; LYC-M, lycopene medium-dose group; LYC-H, lycopene high dose group. **(A)** Motilin (MTL). **(B)** Matrix metalloproteinase-9 (MMP-9). **(C)** Monocyte chemoattractant protein-1 (MCP-1). Values are means standard deviation of 14 mice and triplicate measurements. Different letters indicate significant differences (*P* < 0.05) between different groups.

MTL can regulate gastrointestinal motility and appetite, which also play an important role in regulating the digestion and absorption of nutrients (23). In our study, the concentration of MTL in the IC group was reduced to 0.21-fold that of the NC group, indicating that absolute ethanol inhibits the secretion of motilin (Figure 5A). At the same time, lycopene significantly increased MTL secretion. The concentration of motilin in the LYC group was 1.51-fold that of the NC group, and the concentrations of MTL in the LYC-UL, LYC-L, LYC-M, and LYC-H groups were 3.48-, 4.49-, 3.16-, and 7.16-fold that of IC group, respectively. In addition, we tested the levels of the inflammatory factors MMP-9 and MCP-1. As shown in Figure 5B, the levels of MMP-9 in the IC group was increased to 1.35-fold that of the NC group. But, the MMP-9 in LYC-UL, LYC-L, LYC-H, and LYC-M groups were significantly reduced to 0.52-, 0.62-, 018-, and 0.33-fold that of the IC group, respectively. In addition, the level of MCP-1 in IC group was increased to 1.65-fold that of the NC group (Figure 5C). But the MCP-1 in lycopene experimental groups were significantly lower than that in IC group, suggesting lower levels of inflammatory stimulating factors.

### The Effect of Lycopene on the Levels of CAT, ALT, and AST in Liver Tissues

**Figure 6:**
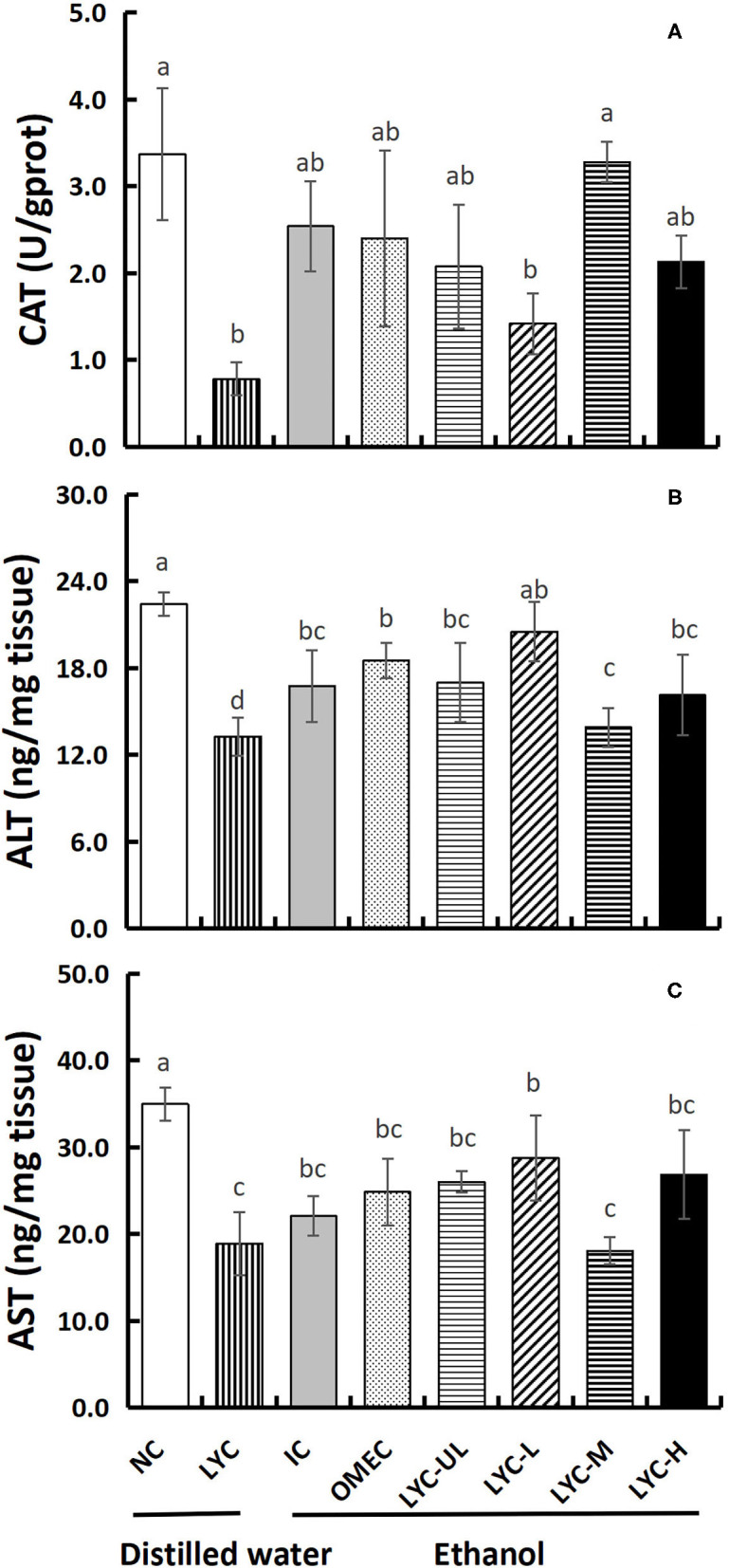
Effect of lycopene on CAT, ALT, and AST of liver tissues. NC, normal control group; LYC, lycopene control group; IC, injury control group; OMEC, omeprazole control group; LYC-UL, ultralow-dose group; LYC-L, lycopene low-dose group; LYC-M, lycopene medium-dose group; LYC-H, lycopene high dose group. **(A)** Catalase (CAT). **(B)** Alanine aminotransferase (ALT). **(C)** Aspartate aminotransferase (AST). Values are means standard deviation of 14 mice and triplicate measurements. Different letters indicate significant differences (*P* < 0.05) between different groups.

In addition, we also tested the levels of the antioxidant enzymes CAT and aminotransferases ALT and AST in liver tissues (Figure 6). Without ethanol treatment, the CAT level in the LYC group was only 0.23-fold that of the NC group. After ethanol treatment, there was no significance between LYC-H and IC groups, but the CAT level in lycopene experimental groups were all higher than LYC group (Figure 6A). The levels of ALT and AST in LYC-M group were significantly reduced to 0.83- and 0.82-fold that of the IC group, respectively (Figures 6B,C). Without ethanol treatment, the levels of ALT and AST in the LYC group were reduced to 0.60- and 0.54-fold of the NC group, respectively. Therefore, the CAT, ALT, and AST levels in the LYC group were all lower than that in NC group without ethanol treatment.

## Discussion

With the continuous increase in risk factors in daily life, gastrointestinal problems have become a global problem. Ethanol is widely considered to be the main risk factor for gastric mucosal damage (24). Lycopene can scavenge free radicals, inhibit acid secretion, and strengthen the gastric mucosal barrier. In particular, lycopene exists in an all-trans configuration under natural conditions, and in this form it is one of the most effective singlet oxygen quenchers in natural carotenoids (24). Thus, does lycopene have a protective effect against gastric mucosal damage caused by ethanol? In this study, an alcohol-induced gastric mucosal injury mouse model was used to investigate the effect of lycopene on gastric mucosal injury. Our results showed that, compared with the normal control, the gastric injury model mice had an obvious pathological phenotype, which manifested as acute gastric mucosal injury. However, the mucosal damage in the lycopene experimental groups, especially the LYC-H group, was more severe than that in the IC group (Figures 1, 2).

Increasing evidence shows that alcohol-induced gastric mucosal damage is closely related to an increase in ROS levels (25). In organisms, SOD can protect the host against ROS-induced lipid peroxidation (26). This study found that, regardless of whether it was induced by ethanol, after supplementation with lycopene, the T-SOD level in the serum of mice increased significantly. It was shown that lycopene can improve the antioxidant capacity of mice. Moreover, in liver tissues, the level of CAT with absolute ethanol treatment in the lycopene experimental group was significantly higher than that without ethanol treatment (LYC group). In addition, NO and MDA are often used to evaluate antioxidant levels in gastric injury models. The levels of NO are closely related to the attack and defense mechanism of the gastric mucosa (27), which can cause the dilation of submucosal arterioles and increase mucosal blood flow in order to buffer the acid entering the lamina propria (28). We found that certain dose of lycopene promoted NO production regardless of ethanol treatment (especially in LYC-H group) (Figure 4), suggesting that lycopene improves the antioxidant capacity of mice and promotes blood flow to counteract the effects of gastric acid. In this study, the amount of gastric juice in the lycopene experimental group was lower than that in the IC group, but the total acidity of the gastric juice in LYC-M and LYC-H groups did not decrease (especially in LYC-H group) (Figure 3). Müller et al. reported that lycopene may increase blood flow and reduce inflammation (29). We hypothesized that lycopene protects against gastric acid by increasing NO levels and increasing blood flow, but does not reduce the amount of gastric acid secreted. In addition, malondialdehyde is one of the end-products of lipid peroxidation and is often used as an indicator to evaluate lipid peroxidation in the gastric mucosa (30, 31). We found that the level of MDA in lycopene experimental groups (especially moderate doses) was significantly lower than that in the IC group after ethanol treatment. This may be due to the antioxidant effect of lycopene and the increase in the T-SOD concentration. Li et al. also found that lycopene enhanced SOD activity and reduced MDA levels in kainic acid-induced mice (32). Therefore, we hypothesized that lycopene could reduce lipid peroxidation by increasing antioxidant enzyme activity, and promote blood flow to resist the effects of gastric acid by increasing the level of NO regardless of ethanol treatment.

However, it can be seen from the macroscopic image (Figure 1) and the tissue morphology image (Figure 2) that supplementation with lycopene (especially at high doses) can aggravate acute gastric mucosal injury induced by absolute ethanol. Although lycopene has strong antioxidant activity, when it is used long-term with alcohol, the effects of lycopene may be more harmful than beneficial. For example, Leo et al. conducted experiments on 14 baboons and found that after taking ethanol, the levels of β-carotene in plasma and liver increased and the levels of retinol decreased (33), indicating that ethanol interfered with conversion of β-carotene to retinol. Conversion of alcohol causes liver damage. That study also pointed out that a deficiency or excess of vitamin A and carotenoids can have harmful effects. Moreover, vitamin A and carotenoids have similar adverse effects in terms of fibrosis, carcinogenesis, and possible embryotoxicity. In a follow-up study, Leo et al. found that liver damage caused by the simultaneous use of β-carotene and ethanol may be related to the effect of ethanol on β-carotene metabolism, which increases vitamin A and retinol, and related to liver toxicity (34). Veeramachaneni et al. found that supplementation with high doses of lycopene can significantly induce the expression of CYP2E1 protein and TNF-α mRNA, and the incidence of inflammatory lesions in the liver of ethanol-fed rat (35). This indicates that there may be a negative synergy between long-term alcohol intake and lycopene supplementation.

In addition, we detected some inflammatory cytokines, including MMP-9 and MCP-1. In the process of alcohol-induced gastric ulcers, the submucosa of the tissue can usually be infiltrated by inflammatory cells. Inflammatory cells are the main source of MMPs (36) and MCP-1 is closely related to inflammation (21). We found that the levels of MMP-9 and MCP-1 did not increase, and no corresponding inflammatory cells were found in the pathological sections. Therefore, acute gastric mucosal injury caused by the simultaneous administration of lycopene and absolute alcohol may have little relationship with inflammatory factors.

ALT and AST are enzymes that catalyze the amino transfer between amino acids and keto acids, which are mainly located in liver tissue cells, and their abnormal elevations often indicate liver cell damage and necrosis (37). In this study, we found that acute intake of a large dose of absolute ethanol after long-term administration of lycopene could aggravate gastric mucosal damage in mice. However, the levels of ALT and AST in LYC-UL, LYC-M, and LYC-H groups did not significantly increase; that is, the damage to liver tissues was not significant (Figure 6). Valerio et al. found that acute alcohol intake affects the synthesis of glucose in the liver (38). Long-term alcoholism causes an increase in liver-related enzymes, such as ALT, AST, and gamma-glutamyl-transpeptidase. Therefore, it is possible that we did not see an increase in the activity of ALT and AST because too few doses of ethanol were administered and due to the short duration of action. In addition, the activities of ALT and AST in the LYC group were significantly lower than those in the NC, OMEC, and IC groups. Knecht et al. found that free radicals can be detected in the bile of a rat alcoholic liver model (39). Lycopene scavenges free radicals. Therefore, the decreased ALT and AST levels in the LYC group in this study may have been an effect of lycopene. Furthermore, lycopene significantly increased MTL secretion regardless of ethanol treatment (Figure 5A), suggesting that lycopene has a protective effect on the stomach and liver without ethanol treatment.

## Conclusions

The study found that lycopene (especially at high doses) aggravates acute gastric mucosal damage caused by absolute ethanol, but this was not due to oxidative stress or inflammatory factors. Lycopene has a protective effect on the stomach and liver without ethanol treatment, which may be a result of its powerful antioxidant activity. Therefore, when alcohol is consumed in large quantities, consumption of lycopene products should be carefully considered. The mechanism of the exacerbation of ethanol induced acute gastric mucosal damage caused by lycopene is still unknown and this will be an important area of future research.

## Data Availability Statement

The original contributions presented in the study are included in the article/Supplementary Material, further inquiries can be directed to the corresponding author.

## Ethics Statement

The animal study was reviewed and approved by Jining Medical University Institutional Animal Care and Use Committee.

## Author Contributions

XC and YaW conceived and designed the experiments, wrote, and revised the paper. XC, YZ, KL, ZL, and XT performed the experiments. XC, YaW, and XF analyzed the data. YuW, NG, and CL assisted in the revision of the manuscript. All authors have read and agreed to the published version of the manuscript.

## Conflict of Interest

The authors declare that the research was conducted in the absence of any commercial or financial relationships that could be construed as a potential conflict of interest.

## Publisher's Note

All claims expressed in this article are solely those of the authors and do not necessarily represent those of their affiliated organizations, or those of the publisher, the editors and the reviewers. Any product that may be evaluated in this article, or claim that may be made by its manufacturer, is not guaranteed or endorsed by the publisher.

## References

[1] Laine L Takeuchi K Tarnawski A. Gastric mucosal defense and cytoprotection: bench to bedside. Gastroenterology. (2008) 135:41–60. 10.1053/j.gastro.2008.05.030 18549814

[2] Dimaline R Varro A. Attack and defence in the gastric epithelium-a delicate balance. Exp Physiol. (2007) 92:591–601. 10.1113/expphysiol.2006.036483 17412751

[3] Melchiorri D Sewerynek E Reiter RJ Ortiz GG Poeggeler B Nisticò G. Suppressive effect of melatonin administration on ethanol-induced gastroduodenal injury in rats in vivo. Br J Pharmacol. (1997) 121:264–70. 10.1038/sj.bjp.0701104 9154336 PMC1564668

[4] Yoo JH Lee JS Lee YS Ku S Lee HJ. Protective effect of bovine milk against HCl and ethanol-induced gastric ulcer in mice. J Dairy Sci. (2018) 101:3758–70. 10.3168/jds.2017-13872 29477532

[5] Pan JS He SZ Xu HZ Zhan XJ Yang XN Xiao HM. Oxidative stress disturbs energy metabolism of mitochondria in ethanol-induced gastric mucosa injury. World J Gastroenterol. (2008) 14:5857–67. 10.3748/wjg.14.5857 18855985 PMC2751896

[6] Arda-Pirincci P Bolkent S Yanardag R. The role of zinc sulfate and metallothionein in protection against ethanol-induced gastric damage in rats. Dig Dis Sci. (2006) 51:2353–60. 10.1007/s10620-006-9301-3 17103035

[7] Yoo JH Park EJ Kim SH Lee HJ. Gastroprotective effects of fermented lotus root against ethanol/HCl- induced gastric uucosal acute toxicity in rats. Nutrients. (2020) 12:808. 10.3390/nu12030808 32204312 PMC7146638

[8] Mun HI Min CK Lee NR Su YS Lee CH. Comparing metabolites and functional properties of various tomatoes using mass spectrometry-based metabolomics approach. Frontiers in Nutrition. (2021) 8:659646. 10.3389/fnut.2021.659646 33898504 PMC8060453

[9] Zhu R Wei J Liu H Liu C Wang L Chen B. Lycopene attenuates body weight gain through induction of browning *via* regulation of peroxisome proliferator-activated receptor gamma in high-fat diet-induced obese mice. J Nutr Biochem. (2020) 78:108335. 10.1016/j.jnutbio.2019.108335 31978713

[10] Rowles JL Ranard KM Smith JW An R Erdman JW Jr. Increased dietary and circulating lycopene are associated with reduced prostate cancer risk: a systematic review and meta-analysis. Prostate Cancer Prostatic Dis. (2017) 20:361–77. 10.1038/pcan.2017.25 28440323

[11] Li L Liu Z Jiang H Mao X. Biotechnological production of lycopene by microorganisms. Appl Microbiol Biotechnol. (2020) 104:10307–24. 10.1007/s00253-020-10967-4 33097966

[12] Di Mascio P Kaiser S Sies H. Lycopene as the most efficient biological carotenoid singlet oxygen quencher. Arch Biochem Biophys. (1989) 274:532–8. 10.1016/0003-9861(89)90467-0 2802626

[13] Boyacioglu M Kum C Sekkin S Yalinkilinc HS Avci H Epikmen ET. The effects of lycopene on DNA damage and oxidative stress on indomethacin-induced gastric ulcer in rats. Clin Nutr. (2016) 35:428–35. 10.1016/j.clnu.2015.03.006 25818123

[14] Jang SH Lim JW Morio T Kim H. Lycopene inhibits *Helicobacter pylori*-induced ATM/ATR-dependent DNA damage response in gastric epithelial AGS cells. Free Radic Biol Med. (2012) 52:607–15. 10.1016/j.freeradbiomed.2011.11.010 22178412

[15] Zhao H Wang Z Ma F Yang X Cheng C Yao L. Protective effect of anthocyanin from *Lonicera Caerulea* var. Edulis on radiation-induced damage in mice. Int J Mol Sci. (2012) 13:11773–82. 10.3390/ijms130911773 23109882 PMC3472774

[16] Li F Lin B Hao Y Li Y Liu J Cong J. Lewis Y promotes growth and adhesion of ovarian carcinoma-derived RMG-I cells by upregulating growth factors. Int J Mol Sci. (2010) 11:3748–59. 10.3390/ijms11103748 21152298 PMC2996800

[17] Zhou J Huang S Wang Z Huang J Xu L Tang Xuefeng. Targeting EZH2 histone methyltransferase activity alleviates experimental intestinal inflammation. Nature Communications. (2019) 10:2427. 10.1038/s41467-019-10176-2 31160593 PMC6547712

[18] Li WF Hao DJ Fan T Huang HM Yao H Niu XF. Protective effect of chelerythrine against ethanol-induced gastric ulcer in mice. Chem Biol Interact. (2014) 208:18–27. 10.1016/j.cbi.2013.11.011 24300194

[19] Wang XY Yin JY Zhao MM Liu SY Nie SP Xie MY. Gastroprotective activity of polysaccharide from Hericium erinaceus against ethanol-induced gastric mucosal lesion and pylorus ligation-induced gastric ulcer, and its antioxidant activities. Carbohydr Polym. (2018) 186:100–9. 10.1016/j.carbpol.2018.01.004 29455967

[20] Róvero Costa M Leite Garcia J Cristina Vágula de Almeida Silva C Junio Togneri Ferron A Valentini Francisqueti-Ferron F. Lycopene modulates pathophysiological processes of non-alcoholic fatty liver disease in obese rats. Antioxidants (Basel). (2019) 8:276. 10.3390/antiox8080276 31387231 PMC6720442

[21] Amirshahrokhi K Khalili AR. Methylsulfonylmethane is effective against gastric mucosal injury. Eur J Pharmacol. (2017) 811:240–8. 10.1016/j.ejphar.2017.06.034 28666801

[22] Xu P Yang L Yuan RY Ye ZY Ye HR Ye M. Structure and preventive effects against ethanol-induced gastric ulcer of an expolysaccharide from *Lachnum* sp. Int J Biol Macromol. (2016) 86:10–7. 10.1016/j.ijbiomac.2016.01.036 26774377

[23] Kitazawa T Kaiya H. Regulation of gastrointestinal motility by motilin and ghrelin in vertebrates. Front Endocrinol (Lausanne). (2019) 10:278. 10.3389/fendo.2019.00278 31156548 PMC6533539

[24] Zhao Z Gong S Wang S Ma C. Effect and mechanism of evodiamine against ethanol-induced gastric ulcer in mice by suppressing Rho/NF-small ka, CyrillicB pathway. Int Immunopharmacol. (2015) 28:588–95. 10.1016/j.intimp.2015.07.030 26225926

[25] Si H Wang FY. Protective effects of bilobalide against ethanol-induced gastric ulcer in vivo/vitro. Biomed Pharmacother. (2017) 85:592–600. 10.1016/j.biopha.2016.11.068 27903426

[26] Liu YH Zhang ZB Zheng YF Chen HM Yu XT Chen XY. Gastroprotective effect of andrographolide sodium bisulfite against indomethacin-induced gastric ulceration in rats. Int Immunopharmacol. (2015) 26:384–91. 10.1016/j.intimp.2015.04.025 25916678

[27] Magierowski M Magierowska K Kwiecien S Brzozowski T. Gaseous mediators nitric oxide and hydrogen sulfide in the mechanism of gastrointestinal integrity, protection and ulcer healing. Molecules. (2015) 20:9099–123. 10.3390/molecules20059099 25996214 PMC6272495

[28] Izzo AA Carlo GD Mascolo N Capasso F. Autore G. Antiulcer effect of flavonoids Role of endogenous PAF. Phytotherapy Res. (1994) 8:179–81. 10.1002/ptr.2650080313

[29] Müller L Caris-Veyrat C Lowe G Böhm V. Lycopene and its antioxidant role in the prevention of cardiovascular diseases-A critical review. Crit Rev Food Sci Nutr. (2016) 56:1868–79. 10.1080/10408398.2013.801827 25675359

[30] Byeon S Oh J Lim JS Lee JS Kim JS. Protective effects of *Dioscorea batatas* flesh and peel extracts against ethanol-induced gastric ulcer in mice. Nutrients. (2018) 10:1680. 10.3390/nu10111680 30400615 PMC6266015

[31] Ma L Liu J. The protective activity of *Conyza blinii* saponin against acute gastric ulcer induced by ethanol. J Ethnopharmacol. (2014) 158:358–63. 10.1016/j.jep.2014.10.052 25446589

[32] Li S Luo Z Lu B Xia S Li C Guan X. Protective effects of lycopene on kainic acid-induced seizures. Epilepsy Res. (2019) 151:1–6. 10.1016/j.eplepsyres.2019.01.010 30669043

[33] Leo MA Kim C Lowe N Lieber CS. Interaction of ethanol with beta-carotene: delayed blood clearance and enhanced hepatotoxicity. Hepatology. (1992) 15:883–91. 10.1002/hep.1840150522 1568731

[34] Leo MA Lieber CS. Alcohol, vitamin A, and beta-carotene: adverse interactions, including hepatotoxicity and carcinogenicity. Am J Clin Nutr. (1999) 69:1071–85. 10.1093/ajcn/69.6.1071 10357725

[35] Veeramachaneni S Ausman LM Choi SW Russell RM Wang XD. High dose lycopene supplementation increases hepatic cytochrome P4502E1 protein and inflammation in alcohol-fed rats. J Nutr. (2008) 138:1329–35. 10.1093/jn/138.7.1329 18567756 PMC2543121

[36] Chakraborty S Stalin S Das N Choudhury ST Ghosh S Swarnakar S. The use of nano-quercetin to arrest mitochondrial damage and MMP-9 upregulation during prevention of gastric inflammation induced by ethanol in rat. Biomaterials. (2012) 33:2991–3001. 10.1016/j.biomaterials.2011.12.037 22257724

[37] Cao YW Jiang Y Zhang DY Wang M Chen WS Su H. Protective effects of *Penthorum chinense Pursh* against chronic ethanol-induced liver injury in mice. J Ethnopharmacol. (2015) 161:92–8. 10.1016/j.jep.2014.12.013 25510733

[38] Valerio LG Jr Parks T Petersen DR. Alcohol mediates increases in hepatic and serum nonheme iron stores in a rat model for alcohol-induced liver injury. Alcoho Clin Exp Res. (1996) 20:1352–61. 10.1111/j.1530-0277.1996.tb01134.x 8947310

[39] Knecht KT Adachi Y Bradford BU Iimuro Y Kadiiska M Xuang QH. Free radical adducts in the bile of rats treated chronically with intragastric alcohol: inhibition by destruction of *Kupffer* cells. Mol Pharmacol. (1995) 47:1028–34. 7746269

