# Supplementary figures and images for: Lycopene Aggravates Acute Gastric Injury Induced by Ethanol
Source: Front Nutr. 2021 Aug 17;8:697879. doi: 10.3389/fnut.2021.697879 (PMC8415829; doi:10.3389/fnut.2021.697879)

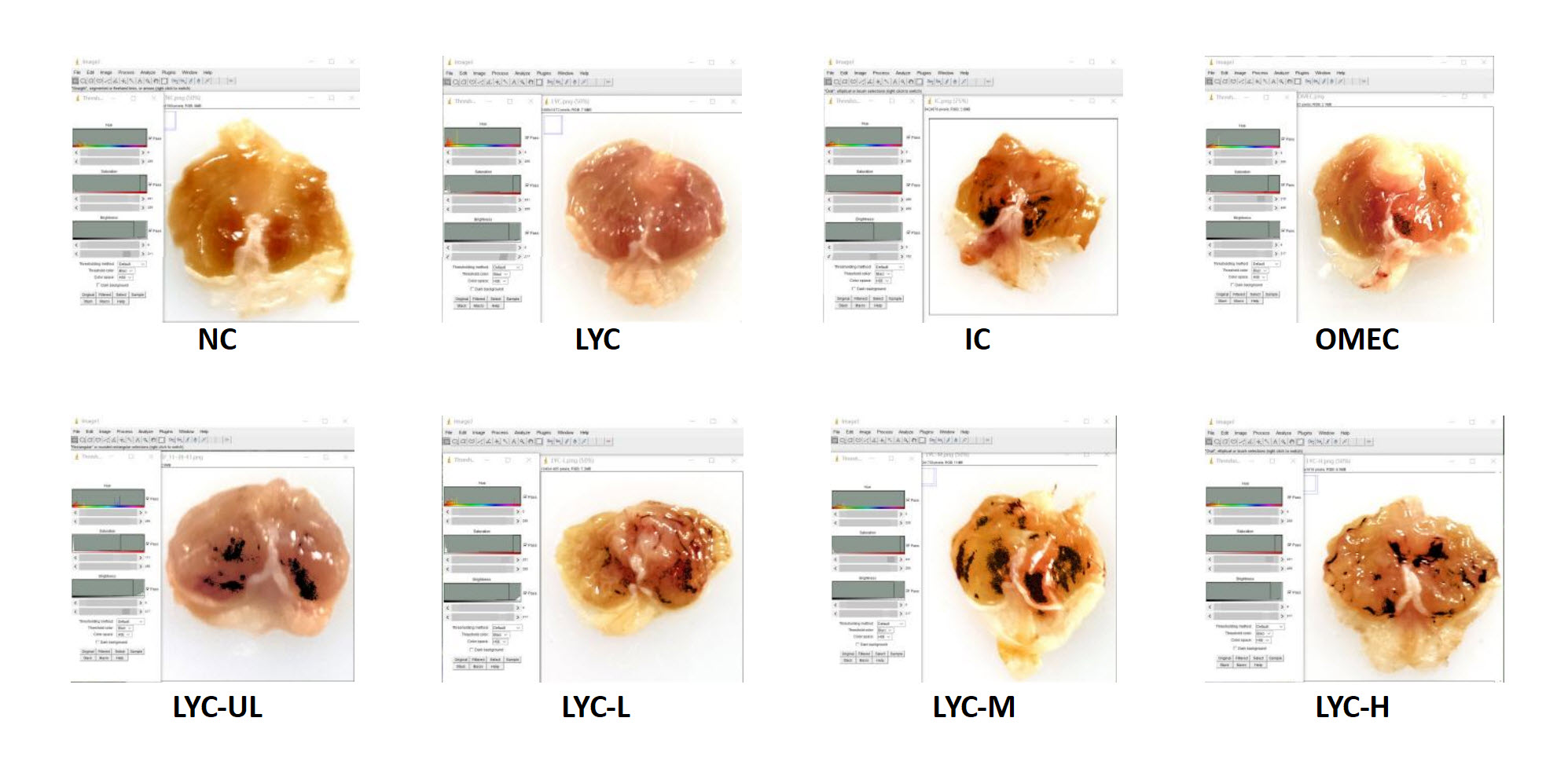

Supplement: Supplementary file 1 [file Image_1.JPEG]
